# LINC00491 Facilitates Tumor Progression of Lung Adenocarcinoma via Wnt/β-Catenin-Signaling Pathway by Regulating MTSS1 Ubiquitination

**DOI:** 10.3390/cells11233737

**Published:** 2022-11-23

**Authors:** Huimin Wan, Tingting Lin, Mengtian Shan, Jingjing Lu, Zhongliang Guo

**Affiliations:** Department of Respiratory and Critical Care Medicine, Shanghai East Hospital, Tongji University School of Medicine, Pudong, Shanghai 200120, China

**Keywords:** lung adenocarcinoma, LINC00491, MTSS1, β-catenin, ubiquitination

## Abstract

**Background:** Long non-coding RNAs have been reported to be involved in tumorigenesis and progression through different regulatory mechanisms. It has been reported that aberrantly expressed long non-coding RNA LINC00491 promotes malignancy in multiple tumors, while the role of LINC00491 in lung adenocarcinoma (LUAD) is little reported and the mechanism for regulating tumor progression has not been elucidated. **Methods:** RNA sequencing and the TCGA database were combined to screen differentially expressed lncRNAs that facilitate tumor progression. The expression level of LINC00491 was examined in LUAD clinical samples and in cell lines using RT-qPCR. In vitro experiments including colony formation assay, EdU assay, cell migration and invasion assay and wound healing assay, and in vivo experiments including xenografting subcutaneous tumors and lung metastasis models were performed to investigate the function of LINC00491 in LUAD tumor progressions. RNA pull-down, mass spectrometry, RIP assays and truncation experiments were carried out to explore the proteins binding to LINC00491 and the specific interactions between the RNA–protein complex. **Results:** Our results showed that LINC0491 was significantly upregulated in LUAD and positively correlated with poor survival. High LINC00491 expression promoted proliferation, migration and invasion, and resulted in a high metastatic burden in LUAD. Using pull-down assay and mass spectrometry, MTSS1 was found binding to LINC00491, and the conducted experiments verified the direct interaction between LINC00491 and MTSS1. Meanwhile, LINC00491 was found to regulate MTSS1 degradation by promoting the MTSS1 ubiquitination level and then activating the Wnt/β-catenin-signaling pathway. LINC00491/MTSS1/β-catenin may act as a complex to facilitate tumor progression. **Conclusions**: In summary, our results found a novel mechanism in which LINC00491 directly interacts with MTSS1 by affecting its ubiquitination modification to promote LUAD proliferation, migration and invasion, then activating the Wnt/β-catenin-signaling pathway, demonstrating its significant role in tumor progression and suggesting that the LINC00491/MTSS1/Wnt/β-catenin-signaling pathway could serve as a potential therapeutic target for lung adenocarcinoma in the future.

## 1. Introduction

Lung cancer has been the second most common malignant cancer and has the highest cancer-associated mortality in the world [1,2]. Approximately 80–85% of lung patients are diagnosed as non-small cell lung cancer (NSCLC) in pathological diagnosis, of which lung adenocarcinoma (LUAD) is the most common subtype [3,4]. Although advances in immunotherapy and chemotherapy have been applied in clinical treatments, recurrence and tumor-related death are still a major challenge due to cancer metastasis [5]. LncRNAs are a special type of transcripts that are longer than 200 nucleotides without a function in the encoding proteins [6,7]. Recent studies on lncRNA have shown that it can regulate cell proliferation, cell cycle, migration and drug resistance [8,9,10]. With the rapid growth of RNA-sequencing techniques, mounting evidence indicates that lncRNAs are involved in gene expression not only at the epigenetic and transcriptional level, but also at posttranscriptional levels [11,12]. To date, several studies have reported that lncRNAs such as MALAT1 and UPLA1 promote tumor proliferation, migration, and metastasis via interacting with the protein in LUAD [10,13]. Thus, as regulators of LUAD tumorigenesis and progression, these aberrant lncRNAs could be potential diagnostic biomarkers and therapeutic targets. Apart from regulating mRNA translation, transport and stability, lncRNAs also regulate protein posttranslational modification and stability [14,15]. Numerous studies indicate that specific RNA-dependent protein interactions play a key role in tumor pathogenesis. For instance, lncRNA STEAP3-AS1 prevents the degradation of STEAP3 mRNA by mediating its m6A modification, which activates Wnt signaling to promote CRC progression [16]. Although various lncRNAs have been extensively studied in LUAD, discovering specific RNA-dependent protein interactions would further provide novel strategies for molecular therapy.

MTSS1, known as missing in metastasis (MIM), is affiliated with the inverse Bin-Amphiphysin-Rvs (I-BAR) family [17]. Previous studies have shown that the expression of MTSS1 dramatically dwindles in some solid tumors, especially metastatic cancers such as colorectal, ovarian and prostate cancer, compared to normal tumors [18,19,20]. Thus, MTSS1 is characterized as a tumor metastasis suppressor protein, which is involved in plasma membrane dynamics, signaling and transcriptional regulation [21,22,23]. However, recent studies found MTSS1 is also highly expressed in some solid tumors such as hepatocellular carcinoma (HCC) [24]. Taylor et al. have reported that low MTSS1 expression in LUAD is closely associated with metastatic burden and poor 5-year survival [25]. However, previous studies on the role of MTSS1 in LUAD are restricted to cells or mouse model, and the regulatory mechanism of MTSS1 is poor.

For this paper, we verified the expression of MTSS1 in large numbers of clinical samples and identified LINC00491, which regulates the ubiquitin–proteasome degradation of MTSS1, resulting in high metastasis and poor prognosis. Further experiments in LUAD cell lines and mouse models confirmed that LINC00491 facilitates cell proliferation, migration and tumor metastasis through Wnt signaling via degrading MTSS1.

## 2. Materials and Methods

### 2.1. Patients and Samples

82 LUAD tissue samples and matched adjacent normal lung tissue samples were collected from February 2015 to March 2019 at Shanghai East Hospital (Tongji University, School of Medicine). The samples were placed in liquid nitrogen immediately after resection. The matched adjacent normal lung tissue samples were collected >3 cm from the edge of tumor without tumor cells. Diagnosis and grading of all cases were confirmed by three experienced pathologists. A summary of the clinicopathological features of LUAD patients is provided in Table S1. The Human Ethics Committee of Shanghai East Hospital at Tongji University (Shanghai, China) has approved this study.

### 2.2. Cell Culture and Transfection

A549, H1299 and H1975 human ADC cell lines and BEAS-2B human bronchial epithelial cells were obtained from the Chinese Academy of Science (Shanghai, China). These cell lines were cultured in complete Dulbecco’s modified Eagle’s medium (DMEM) containing 15% fetal bovine serum (Gibco) and 1% penicillin-streptomycin solution (Gibco), under 5% CO2 and at 37 °C in a chamber.

Lentiviral constructs overexpressing *LINC00491* and *MTSS1* were designed and synthesized by GenePharma Co., Ltd. (Shanghai, China). ShRNA plasmids for targeting *LINC00491* together with their nonspecific control shRNA, and plasmids targeting LINC00491 FL, LINC00491 ∆1, LINC00491 ∆2 and LINC00491 ∆3 were designed and purchased by GenePharma Co., Ltd. After transfection for 48 h, the transfection efficiency of cells was evaluated using RT-qPCR. The sequences of shRNAs were as follows: LINC00491-sh1, 5′-GGATATGTGCAGGGAGTCTAG-3′; LINC00491-sh2, 5′-GGTGTATTCCACATT GTCTCT-3′.

RNA isolation, reverse transcription and quantitative reverse transcription–polymerase chain reaction (RT-qPCR) followed.

RNA from cell lines and tissue samples was extracted using Trizol reagent (Invitrogen, Camarillo, CA, USA) according to the manufacturer’s instructions. The RNA from the cytoplasm contents and nucleus was isolated and extracted utilizing a PARISTM Kit (Thermo Fisher, MA, USA). Then, RNA samples were reverse-transcribed into cDNA using a HiScript^®^ II 1st Strand cDNA Synthesis Kit (Enzyme, Nanjing, China). The cDNA templates were amplified by RT-qPCR using a SYBR Green Master Mix (Enzyme, Nanjing, China). GAPDH was applied as an internal control. The primer sequences used were as follows: LINC00491-F, 5′- AAATACCACCACCCCATC-3′; LINC00491-R, 5′- GGAAACATTCTGCCTCCT-3′; MTSS1-F, 5′-CAGTCCCAGCTTCGGACAAC-3′; MTSS1-R, 5′- TGAGAGCAGATCCAATCTCCC-3′; GAPDH-F, 5′-GAGTCAACGGATTTGGTCGT-3′; GAPDH-R, 5′-TTGATTTTGGAGGGATCTCG-3′. The 2^−∆∆CT^ method was adopted to analyze the relative expression.

### 2.3. Colony Formation Assay

A total of 1500 cells were seeded per well in 6-well plates and the culture medium was refreshed every 4 days. After 2 weeks, the colony cells were fixed using 4% paraformaldehyde for 20 min at room temperature and then stained with 0.1% crystal violet for 30 min. The number and size of clones were evaluated to compare the proliferation ability of LUAD cells in groups.

### 2.4. Cell Migration and Invasion Assay

A total of 5 × 10^4^ cells were seeded into the upper chambers of transwell inserts (Corning, Cambridge, MA, USA) with or without Matrigel (Corning) containing 200 µL of serum-free DMEM, and 500 µL DMEM containing 10% FBS was put in the lower chambers. After 24 h, the filters were washed with PBS three times, fixed with 4% paraformaldehyde, and then stained with crystal violet. The number of cells was counted to evaluate the migration and invasion ability of cells.

### 2.5. Wound Healing Assay

Monolayers were scratched with a 200-µL pipette tip across the center of wells when cells reached 90% confluence. Then, cells were cultured in the serum-free medium and imaged at 24 h intervals to record wound width. ImageJ software was adopted to quantify the wound width.

### 2.6. RNA-Binding Protein Immunoprecipitation Assay 

The RIP assay was performed as previously described. In brief, cells were collected using an RIP lysis buffer upon reaching 90% confluence. In the meantime, a human anti-MTSS1 antibody was incubated with magnetic beads to conjugate the antibody to the magnetic beads. Collected using a magnetic separator, proteinase K was added to digest the protein. Finally, the Trizol reagent was used to extract supernatants containing RNA. Normal rabbit IgG in the RIP Kit was used as a negative control.

### 2.7. RNA Pull-Down Assay

A biotin-labelled LINC00491 mimic was synthesized and transfected into cells as previously reported [26]. Cells were collected after 24 h and incubated at 4 °C for 4 h with M-280 streptavidin magnetic beads (Invitrogen). After a 4-h incubation, lysis buffer containing 10% SDS and proteinase K were used to wash the beads, and then supernatants were collected. RNA was isolated by combining acid-phenol: chloroform with collected supernatants. PCR assays were performed to detect the coprecipitated RNA.

### 2.8. 5-Ethynyl-2′deoxyuridine (EdU) Assay

The EdU assay was performed using a Cell-light TM EdU DNA cell proliferation kit (RiboBio, Guangzhou, China) according to the manufacturer’s instructions. Briefly, A549 and H1299 cells (1 × 10^3^ per well) were seeded into 96-well plates. After 24 h, the LUAD cells were incubated with 50 µM EdU under 5% CO_2_ and at 37 °C in a chamber for 2 h, then fixed with 4% paraformaldehyde for 15 min and permeabilized with Triton X-100. Finally, Apollo Dye Solution was used to stain the cells and Hoechst 33,342 was used to stain the nucleic acids. Images were acquired on the basis of a Leica SP5 confocal microscope (Leica Microsystems, Wetzlar, Germany).

### 2.9. RNA Fluorescence In Situ Hybridization (FISH) 

The fluorescent probes of LINC00491 were synthesized using Servicebio (Wuhan, China). Cells were fixed using 4% paraformaldehyde, and then 0.25% Triton X-100 was used to permeabilize cells. Then, LUAD cells were hybridized with labeled probes specific to LINC00491 in a reaction buffer at 37 °C overnight. Images were acquired on the basis of a Leica SP5 confocal microscope (Leica Microsystems, Wetzlar, Germany).

### 2.10. ISH (In Situ Hybridization)

The digoxigenin (DIG)-labeled LINC00491 probes were purchased from Servicebio (Wuhan, China). In short, the paraffin-embedded LUAD samples were deparaffinized in xylene and rehydrated via graded ethanol. Pepsin was used to digest sections for 20 min, and then sections were prehybridized overnight at 40 °C. After washing, the sections were incubated in a LINC00491-specific probe along with subsequently biotinylated mouse anti-DIG-HRP antibody, and further stained with biotinylated peroxidase. Finally, LINC00491 expression was visualized using 3,3′-diaminobenzidine (DAB) substrate and hematoxylin was used as a counterstain. 

### 2.11. Immunofluorescence (IF) Assay 

2 × 10^4^ LUAD cells were seeded in confocal dishes (NEST, Wuxi, China). After 24 h, the cells were fixed, permeabilized and blocked for 2 h. Then the cells were incubated with primary antibodies at 4 °C for 8 h, and with secondary antibodies for another 1 h at room temperature. Then, DAPI was used to stain the nuclei. Finally, a Leica SP5 confocal microscope (Leica Microsystems, Wetzlar, Germany) was adopted to acquire images. 

### 2.12. Western Blot Analysis 

The whole protein from cells and tissues was extracted with a RIPA buffer (Beyotime, Shanghai, China). The protein extracts were incubated with the corresponding antibody at 4 °C overnight after being subjected to 10% SDS-PAGE, then transferred to polyvinylidene fluoride (PVDF) membranes (Millipore). After being washed with TBST (TBS + Tween) three times, the membranes were incubated with goat anti-rabbit IgG (H+L) or goat anti-mouse IgG (H+L) antibodies for 1 h. Enhanced chemiluminescence (ECL) (Millipore, Burlington, MA, USA) was adopted to visualize the protein gel. β-actin served as an internal control. Primary antibodies in the experiment are listed in Table S3.

### 2.13. Hematoxylin-Eosin (HE) and Immunohistochemistry (IHC) Analysis

HE staining was conducted according to the manufacturer’s instructions using a hematoxylin-eosin staining kit (Beyotime, Shanghai, China). For IHC staining, sections of tissue samples were incubated with the corresponding antibody overnight at 4 °C, then with HRP Polymer secondary antibodies at room temperature for 1 h. Finally, chromogen (Servicebio, Wuhan, China) was taken to the stain sections, and then images were gained in the microscope for analysis.

### 2.14. Denature-IP Assay

All the ubiquitin assays were performed in denaturing conditions. IP assays were carried out according to the manufacturer’s instructions using a Magnetic IP/Co-IP Kit (Thermo Fisher, Waltham, MA, USA). Briefly, cells were lysed in an NP-40 lysis buffer (Beyotime) and the lysates were centrifuged. Incubation of the above supernatant and the targeted antibody was assigned with rotation at 4 °C overnight. After being added to samples on a rotary shaker at 4 °C for 6–8 h, 1 mL wash buffer containing 0.08% NP-40, 150 mM NaCl, 50 mM Tris-HCl (pH 8.0) and 5 mM MgCl_2_ was used to wash protein A/G magnetic beads at 4 °C for 15 min three times. Finally, protein A/G magnetic beads were transferred to a 2X SDS-PAGE sample loading buffer (Beyotime) and incubated at 95 °C for 9 min. Co-immunoprecipitated (co-IP) proteins were analyzed using immunoblot procedures. 

### 2.15. Xenografting Subcutaneous Tumor and Lung Metastasis

4 × 10^6^ logarithmically growing A549 cells from different groups were injected subcutaneously into the flank of nude mice (female, 4-week-old, *n* = 6 per group) within 100 µL PBS. The weight and volume of tumors were examined every 5 days. After 3 weeks, euthanasia was performed on mice, and the xenografting tumors were collected for IHC analysis.

For the lung metastasis model, 1 × 10^6^ logarithmically growing A549 cells from different groups were injected via tail vein (6 mice per group). After 8 weeks, euthanasia was conducted in mice and the metastatic lung tissues were collected for HE.

### 2.16. Statistical Analysis

Statistical graphs in this study were created using GraphPad Prism 8.0 software (GraphPad Software Inc., San Diego, CA, USA). All statistical data are expressed as the means ± S.D. Comparisons between groups were examined using two-tailed Student’s *t*-test or one-way ANOVA. Expression correlation was evaluated by means of Pearson’s coefficient correlation. The Kaplan–Meier method was adopted to plot the survival curve and the comparison was analyzed using log-rank tests. *p* value < 0.05 indicates statistical significance.

## 3. Results

### 3.1. LINC00491 Is Specifically Upregulated in LUAD and Correlated with Poor Survival

**Figure 1:**
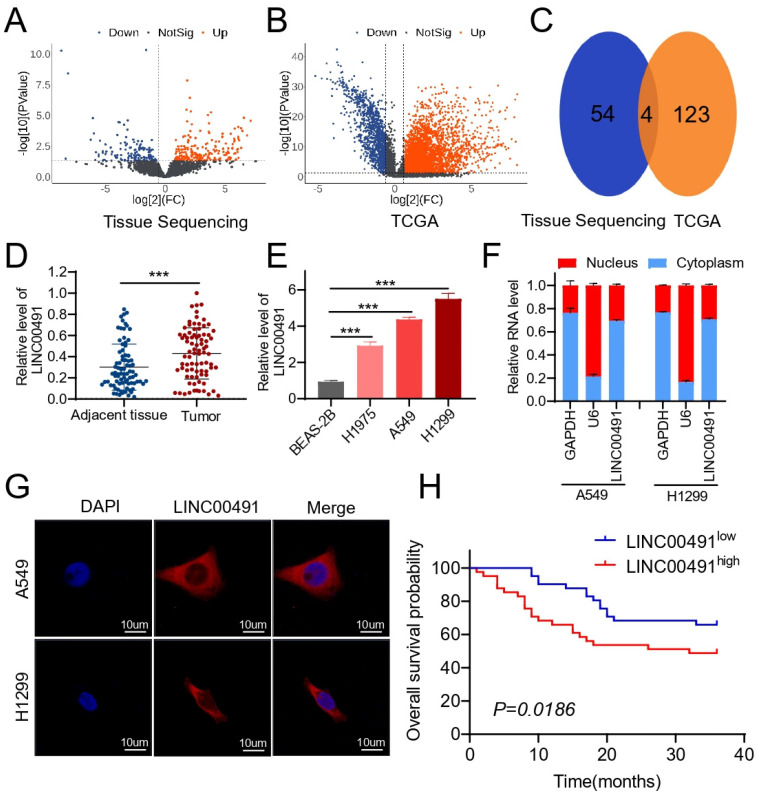
Global analyses of aberrant lncRNAs in LUAD and high-LINC00491 expression in LUAD tissues and cell lines. (**A**) The volcanic plot of lncRNAs between LUAD and match adjacent normal tissues. (**B**) A volcanic plot of lncRNAs between LUAD and normal lung tissues in the TCGA database. (**C**) The common, significantly upregulated lncRNAs between tissue sequencing and the TCGA database in LUAD. (**D**) Detection of the LINC00491 level in the isolated RNA from the cytoplasm and cell nucleus in A549 and H1299 cells using RT-qPCR assays. (**E**) Analysis of LINC00491 expressions in A549, H1299 and H1975 cells relative to that in BEAS-2B cells using RT-qPCR. (**F**) The relative expression of LINC00491 in the cytoplasm and nucleus using RT-qPCR assays. (**G**) Localization of LINC00491 using FISH assay in A549 and H1299 cells. Scale bar = 10 µm. (**H**) The log-rank test of the impact of the LINC00491 expression level on the survival of patients with LUAD. In each experiment, three replicates were conducted. Data are presented as the mean ± SD. ***, *p* < 0.001.

To investigate the potential oncogenic lncRNAs in LUAD, three pairs of LUAD and matched adjacent normal lung tissues were analyzed based on RNA sequencing; the basic information of patients is exhibited in Table S2. As shown in Figure 1A, among 4684 lncRNA transcripts identified from the sequencing data, 134 and 91 lncRNAs were differentially up- and down-regulated in the cohort. Further analysis was performed on LUAD and normal lung tissues from The Cancer Genome Atlas (TCGA) database (Figure 1B). When the significant differences between LUAD and normal lung tissues were assigned at a ≥5 log2-fold change and *p*-value of <0.05, 54 and 123 lncRNAs were differentially expressed in tissue sequence and in the TCGA (Figure 1C). Among the four common upregulated lncRNAs, the difference of LINC00491 was most significant. To further confirm the expression of LINC00491, an RT-qPCR assay was conducted in 82 pairs of LUAD and matched normal lung tissues, which showed a consistent trend of LINC00491 in expression levels as it appeared in the sequencing data (Figure 1D). Then, the expression of LINC00491 was examined in normal lung bronchial epithelial cells (BEAS-2B) and LUAD cell lines (A549, H1299 and H1975) using RT-qPCR; the data indicated that LINC00491 was obviously upregulated in A549, H1975 and H1299 cells relative to BEAS-2B cells (Figure 1E). Consequently, A549 and H1299 cell lines were further investigated. Above all, these results confirmed that LINC00491 was significantly up-regulated both in LUAD cells and tissues.

What is more, RT-qPCR assays and RNA FISH assays were performed to clear the location of LINC00491 in the cell, and the results showed that LINC00491 was largely focalized in the cytoplasm of A549 and H1299, amplified with RNA isolated from the cell nucleus and cytoplasmic contents (Figure 1F,G). Subsequently, the log-rank test was adopted to evaluate the relevance between the LINC00491 expression level and survival in LUAD patients. As shown in Figure 1H, patients with higher LINC00491 expression levels exhibited a worse three-year overall survival (OS) (*p* = 0.0186) compared with those with lower LINC00491 expression levels. Taken together, the above results illustrate that LINC00491 was differentially up-regulated in LUAD patients and LINC00491 expressional levels could be an independent prognostic factor for predicting clinical outcomes in patients with LUAD.

### 3.2. LINC00491 Promotes LUAD Cell Proliferation, Invasion, Migration and Metastasis In Vitro and In Vivo

**Figure 2:**
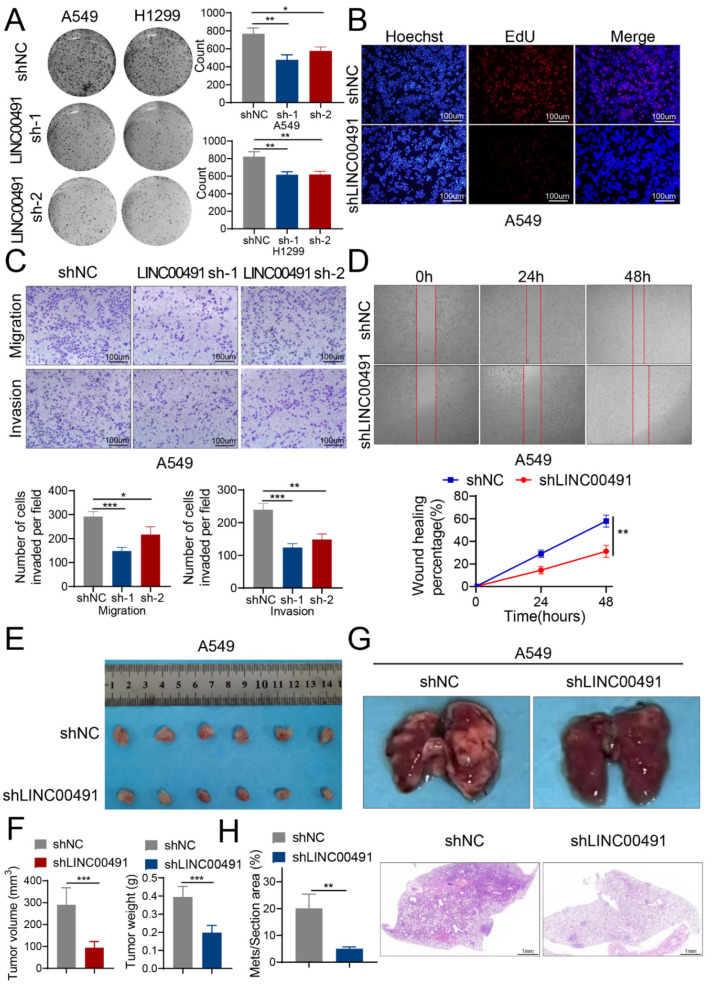
Knockdown of LINC00491 suppresses the proliferation, migration and invasion of LUAD in vitro and in vivo. (**A**) Representative images of colony formation assays in shNC and LINC00491 sh-1 and sh-2 groups (left panel). Quantification of results is exhibited in the right panel. (**B**) The proliferation capability was measured using EdU assays in shNC and LINC00491 sh-1 and sh-2 groups. Scale bars = 100 µm. (**C**) Transwell assays analyzed the migrative and invasive capacity of A549 cells in shNC and LINC00491 sh-1 and sh-2 groups (upper panel). Quantification of results is exhibited in the bottom panel. Scale bars = 100 µm. (**D**) Representative images of the scratch wound healing assay in shNC and shLINC00491 groups (upper panel). Quantification of results is exhibited in the bottom panel. (**E**) Representative images of xenograft tumors in nude mice from the shNC and shLINC00491 groups (*n* = 6). (**F**) Analysis of the tumor volume and tumor weight in the shNC and shLINC00491 groups. (**G**) Representative images of lung metastases in nude mice from the shNC and shLINC00491 groups (upper panel). HE staining of lung metastases in the shNC and shLINC00491 groups (bottom panel). Scale bars = 1 mm. (**H**) Quantification of the percentage of metastatic area is exhibited in the left panel. In each experiment, three replicates were conducted. Data are presented as the mean ± SD. *, *p* < 0.05; **, *p* < 0.01; ***, *p* < 0.001.

To identify the role of LINC00491 in LUAD, short hairpin RNA (shRNA) targeting LINC00491 was transfected in A549 and H1299 to silence LINC00491 and the transfection efficiency was confirmed using RT-qPCR (Figure S1A). Colony formation and EdU assays exhibited that a knockdown of LINC00491 strikingly suppressed the colony formation efficiency and proliferative ability of LUAD cells (Figure 2A,B and Figure S2A). Transwell and wound healing assays indicated that the invasion and migration capability of the two cell lines in the shLINC00491 group was weakened compared to the shNC group (Figure 2C,D and Figure S2B,C). To further explore the in vivo role of LINC00491, a xenograft tumor model was adopted by subcutaneously injecting shNC or shLINC00491 A549 cells into the flanks of nude mice (Figure 2E). The results reflected that the silence of LINC00491 obviously alleviated the xenograft volume and weight in comparison with the shNC group (Figure 2F). Furthermore, to construct the metastatic mouse model, shLINC00491 or shNCA549 cells were injected into the caudal vein of nude mice. The knockdown of LINC00491 markedly attenuated the number of the spread nodules in the pulmonary region, and haematoxylin and eosin (HE) staining confirmed the metastasis foci (Figure 2G,H). Consistent with the conclusion of the xenograft tumor growth experiments, the silence of LINC00491 dramatically decreased the lung metastasis capability of LUAD cells.

### 3.3. LINC00491 Interacts with MTSS1 Protein by Reducing Its Stability in LUAD Cells

**Figure 3:**
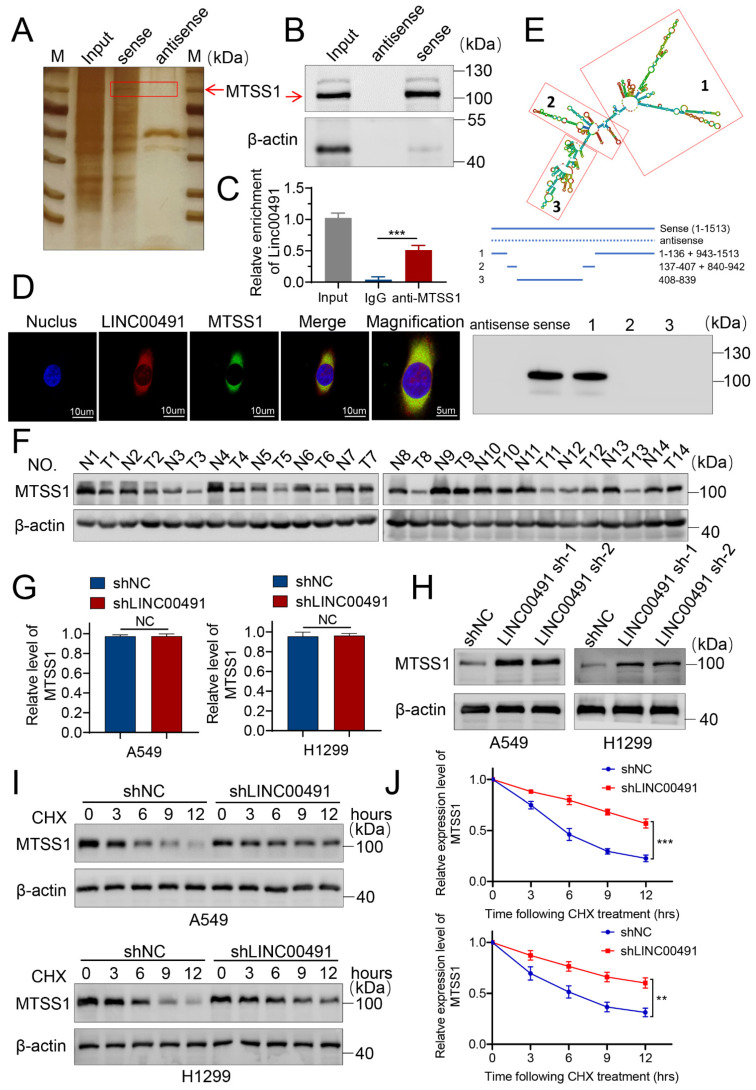
LINC00491 suppresses the expression of the MTSS1 protein by enhancing its degradation. (**A**) Representative silver-stained gel of proteins pulled down by the sense and antisense of LINC00491. Mass spectrum was used to identify differentially exhibited lanes. (**B**) Western blot analysis of the pulldown product MTSS1. (**C**) RT-qPCR analysis of RIP. (**D**) Representative images of the colocalization of LINC00491 with MTSS1 proteins were obtained using a confocal microscope, detected by FISH and IF assays in A549 cells. (**E**) Schematic diagram shows the domain truncation mutants of the LINC00491 sense strand (upper panel). Western blot (bottom panel) showed the MTSS1 protein in the immunoprecipitated complex pulled down by LINC00491 and its truncations. (**F**) Expression of MTSS1 in LUAD and matched normal lung tissues. (**G**) The expression levels of MTSS1 were evaluated in the shNC and shLINC00491 groups of A549 and H1299 using RT-qPCR. (**H**) The expression levels of MTSS1 protein were analyzed in the shNC and shLINC00491 groups of A549 and H1299. (**I**) Immunoblot detection of MTSS1 in the shNC and shLINC00491 groups treated with 25 µg/mL CHX for 0, 3, 6, 9, and 12 h. (**J**) Analysis of the MTSS1 degradation rate. In each experiment, three replicates were conducted. Data are presented as the mean ± SD. **, *p* < 0.01; ***, *p* < 0.001.

To explore the potential mechanism by which LINC00491 exerts its biological functions in LUAD cells, RNA pull-down assays followed by mass spectrometry (MS) were performed. Silver staining showed a protein was markedly focused around 105 kDa compared with the LINC00491 antisense (Figure 3A). The following mass spectrometry assays indicated MTSS1 was a potential protein interacting with LINC00491 in LUAD cells (Figure S3A). Subsequently, an immunoblot assay confirmed the existence of MTSS1 in the LINC00491 pull-down protein complex (Figure 3B). A further RIP assay was carried out to prove the interaction between LINC00491 and MTSS1 using antibodies against MTSS1 (Figure 3C). The result revealed that MTSS1 was directly bound to LINC00491. Moreover, the MTSS1 protein was confirmed as colocalized with LINC00491 in the cytoplasm of A549 and H1299 cells (Figure 3D and Figure S3B). Previous studies found that lncRNAs function mainly depending on linear sequences rather than conserved secondary structures. To investigate the specific region of LINC00491 bound to MTSS1, three truncation mutants (1–136 nt + 943–1513 nt, 137–407 nt + 840–942 nt and 408–839 nt) of LINC00491 were synthesized to carry out RNA pull-down assays. As can be seen in Figure 3E, 1–136 nt + 943–1513 nt of LINC00491 showed a strong affinity for the MTSS1 protein. Collectively, the above results suggest that LINC00491 and MTSS1 could interact as an RNP complex in vitro, and 1–136 nt + 943–1513 nt of LINC00491 presented strong affinity for the MTSS1 protein.

Previous studies have proved that MTSS1 could function as a tumor suppressor in a number of cancers including colorectal, ovarian and breast cancer [27,28,29]. However, few accounts of the mechanism of lncRNA and MTSS1 were reported in LUAD. To uncover the function of MTSS1 in LUAD, samples of LUAD and adjacent normal tissue were collected to analyze the MTSS1 protein level. The data revealed that MTSS1 was significantly down-regulated in LUAD samples (Figure 3F). Then, we sought to explore the regulatory role of LINC00491 on the MTSS1 protein. The knockdown of LINC00491 elevated MTSS1 protein levels in A549 and H1299 cells, but had no influence on the mRNA level (Figure 3G,H). To further determine the mechanism affecting the MTSS1 protein level, cycloheximide (CHX) was used to examine whether LINC00491 affects the translation of MTSS1 protein in LUAD cells (Figure 3I,J). The LINC00491 knockout prolonged the half-life of MTSS1 in the protein level of A549 and H1299 cells after CHX treatment for 3, 6, 9 and 12 h, suggesting that LINC00491 affects MTSS1 protein at the posttranslational level instead of the translational level.

### 3.4. LINC00491 Promotes MTSS1 Degradation via the Ubiquitin–Proteasome Degradation through Wnt/β-Catenin-Signaling Pathway 

**Figure 4:**
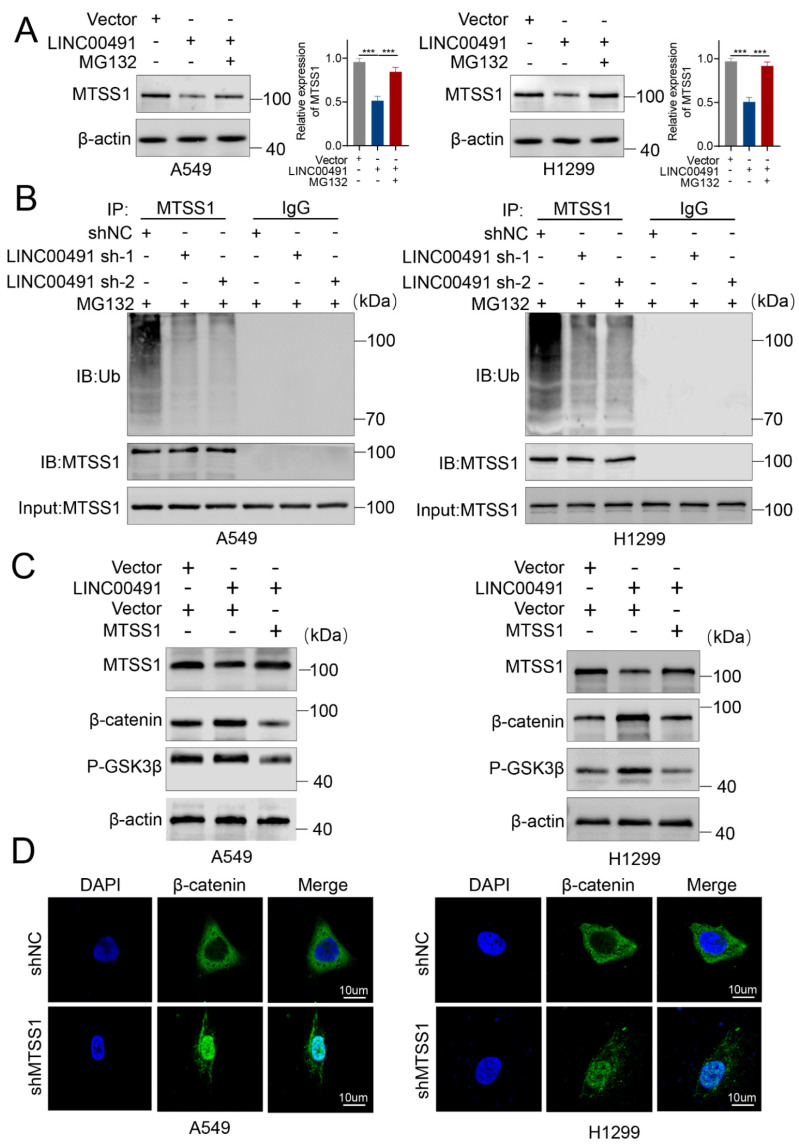
LINC00491 activates the Wnt-signaling pathway via promoting the ubiquitin–proteasome degradation of MTSS1. (**A**) Western blot showing MTSS1 protein in LINC00491 overexpressed LUAD cells treated with MG132 (5 µM) for 24 h. Quantification of results is exhibited in the right panel. (**B**) Ubiquitinated MTSS1 immunoprecipitated with anti-MTSS1 antibody or IgG control and immunoblotted with an anti-ubiquitin antibody in LUAD cells in the shNC and shLINC00491 groups treated with MG132. (**C**) Western blot analysis of Wnt/β-catenin and p-GSK3β protein expression in A549 and H1299 cells transfected with LINC00491, and/or MTSS1. Mock plasmids served as control. (**D**) Representative images of immunofluorescence indicating the expression and subcellular localization of β-catenin (green) in LUAD cells transfected with shNC, or shMTSS1. The nucleus was counterstained with DAPI (blue). In each experiment, three replicates were conducted. Data are presented as the mean ± SD. ***, *p* < 0.001.

What’s more, the enforced expression of LINC00491 dramatically reduced the MTSS1 protein expression, while it was reversed following treatment with MG132 (Figure 4A). These results suggest that LINC00491 promotes the degradation of the MTSS1 protein.

The ubiquitin-proteasome pathway is a key posttranslational modification resulting in the degradation of proteins. To explore whether LINC00491 decreases the MTSS1 protein level through ubiquitin–proteasomal degradation, denature-IP experiments were carried out to examine the ubiquitination level of MTSS1 when LINC00491 was knocked down or not in A549 and H1299 cell lines (Figure 4B). The silence of LINC00491 significantly decreased the ubiquitinated MTSS1 and then increased the MTSS1 protein level, suggesting that LINC00491 enhances MTSS1 ubiquitination and down-regulates MTSS1 in LUAD cell lines. 

Yang et al. revealed the activation of Wnt/β-catenin-signaling attributes in tumor growth and metastasis in lung cancer [30]. Previous studies reported that MTSS1 exerts its biological functions through the Wnt-signaling pathway. Wu et al. reported that MTSS1 inhibits the Wnt-signaling pathway in ovarian cancer, while Chen et al. found that MTSS1 activates the Wnt-signaling pathway in osteogenic differentiation [19,31]. To clarify whether LINC00491 promotes LUAD proliferation and metastasis through the Wnt-signaling pathway via degrading MTSS1, LINC00491 was overexpressed in LUAD cell lines. And the results showed that MTSS1 protein level was impaired, while the Wnt-signaling pathway was activated (Figure 4C). Nevertheless, an enforced expression of MTSS1 reversed the upregulation of the Wnt-signaling pathway activated by LINC00491. Furthermore, Lithium chloride (LiCl), as the agonist of the Wnt-signaling pathway by inhibiting GSK3β activity and then leading to the stabilization of β-catenin, was used to counteract the effects of MTSS1 on the Wnt signaling [19,32,33]. The result showed that LiCl restored the expression level of β-catenin and that the p-GSK3β protein was decreased by the LINC00491 knockdown (Figure S4A). Transwell assays indicated that the invasion and migration capability decreased by the LINC00491 knockdown was reversed by LiCl (Figure S4B). Additionally, the results of confocal immunostaining suggested that upon knockdown of MTSS1, more β-catenin was translocated to the nucleus (Figure 4D). Above all, these results indicate that LINC00491 promotes the ubiquitination of MTTS1 for regulating Wnt signaling.

### 3.5. LINC00491 Promotes LUAD Proliferation and Metastasis via Degrading MTSS1 

**Figure 5:**
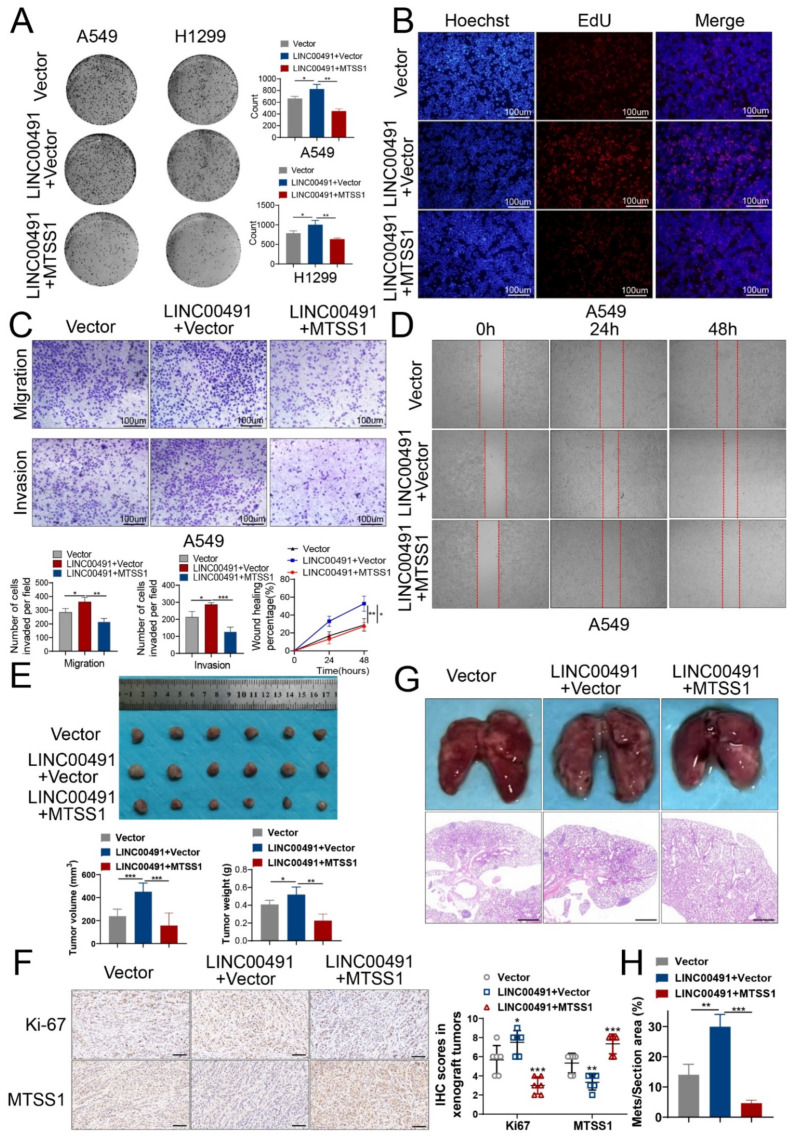
MTSS1 reverses the effects of LINC00491 on LUAD proliferation, migration, invasion and metastasis in vitro and in vivo. (**A**) Representative images of colony formation assays indicated that the overexpression of MTSS1 reversed the growth promotion effect of LINC00491’s overexpression in LUAD cells. Quantification of results is exhibited in the right panel. (**B**) The proliferation ability measured by EdU assays showed that overexpression of MTSS1 reversed the growth promotion effect of LINC00491’s overexpression in A549 cells. Scale bars = 100 µm. (**C**) Transwell assays showed that the overexpression of MTSS1 reversed the migrative and invasive capacity promoted by LINC00491’s overexpression in A549 cells (upper panel). Quantification of results is exhibited in the bottom panel. Scale bars = 100 µm. (**D**) Representative images of the scratch wound healing assay showed that the overexpression of MTSS1 reversed the migrative capacity promoted by LINC00491’s overexpression in A549 cells. Quantification of results is exhibited in the bottom panel. (**E**) Representative images of xenograft tumors in nude mice from mock plasmids, LINC00491 and mock plasmids, and LINC00491 and MTSS1 groups (*n* = 6), the analysis of tumor volume and weight is on the bottom. (**F**) IHC of Ki67 and MTSS1 in xenograft tumors (left panel). Analysis of IHC was in the right panel. Scale bars = 50 µm. (**G**) Representative images of lung metastases in nude mice from mock plasmids, LINC00491 and mock plasmids, and LINC00491 and MTSS1 groups (upper panel). HE staining of lung metastases is in the bottom panel. Scale bars = 100 µm. (**H**) Analysis of metastatic area in the HE staining. In each experiment, three replicates were conducted. Data are presented as the mean ± SD. *, *p* < 0.05; **, *p* < 0.01; ***, *p* < 0.001.

We next further investigate whether LINC00491 interacts with MTSS1 to promote the malignance of the LUAD cell. Colony formation and EdU assays showed that the overexpression of MTSS1 dramatically attenuated the proliferation capability promoted by LINC00491’s overexpression (Figure 5A,B and Figure S5A). Transwell and wound healing assays indicated that the invasion and migration capability of the two cell lines strengthened by LINC00491 overexpression was also weakened when MTSS1 was overexpressed (Figure 5C,D and Figure S5B,C). 

Additionally, for in vivo experiments, the xenograft tumor model verified that the overexpression of MTSS1 reversed the proliferation ability of the LUAD cell in vivo, enhanced by LINC00491’s overexpression (Figure 5E). The IHC staining of MTSS1 and Ki67 verified that LINC00491 promoted LUAD proliferation via regulating MTSS1 protein expression (Figure 5F). The metastatic mouse model also revealed that MTSS1 could reverse the metastasis capability induced by LINC00491’s overexpression in LUAD, and HE staining of metastatic lung regions was consistent with the trend above (Figure 5G,H). 

### 3.6. High LINC00491 Levels and Low MTSS1 Protein Levels Are Correlated with Poor Survival Rates of Patients with LUAD

**Figure 6:**
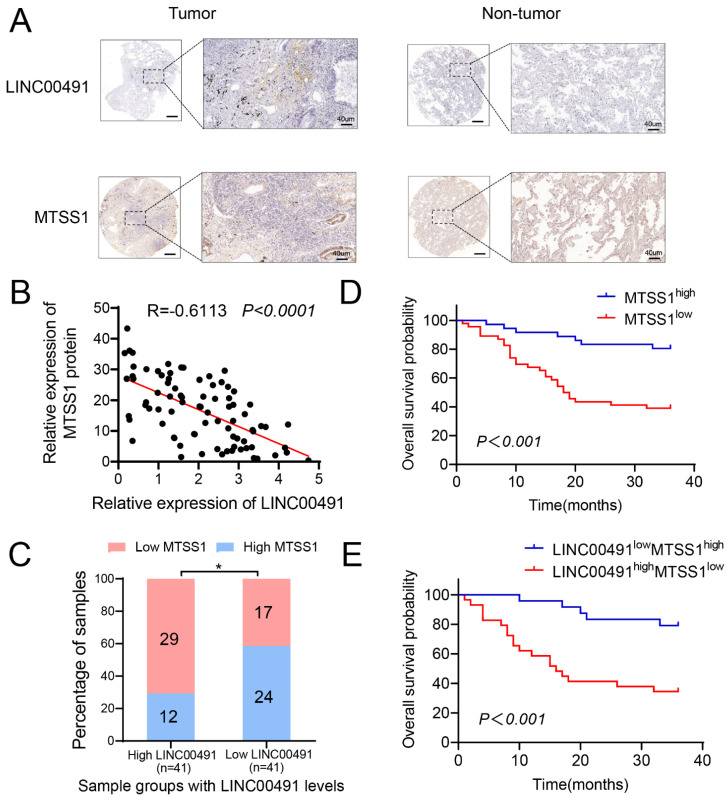
LINC00491 and MTSS1 were associated with the prognosis of LUAD patients. (**A**) Representative images of ISH and IHC assay showed LINC00491 and MTSS1 expression in LUAD and matched normal lung tissues. Scale bars = 200 µm and 40 µm. (**B**) Correlation between LINC00491 and MTSS1 protein expression in LUAD patients. (**C**) Positive correlation of LINC00491 with MTSS1 protein levels. The 82 samples were classified into two groups (Low USP32: 41; High USP32: 41). (**D**) The log-rank test of the impact of the MTSS1 protein level on the survival of patients with LUAD. (**E**) Retrospective analysis of Kaplan–Meier plots for LINC00491 and MTSS1 expression in association with overall survival time. Data are presented as the mean ± SD. *, *p* < 0.05.

To explore the clinical significance of the LINC00491–MTSS1 complex in LUAD, the levels of LINC00491 and MTSS1 were measured in LUAD samples. As shown in Figure 6A, LINC00491 positive rates were considerably increased in LUAD compared to adjacent normal lung tissues, while MTSS1 expression was decreased. Meanwhile, Pearson’s correlation proved the inverse correlation between the expression of LINC00491 and the protein level of MTSS1 both in LUAD and match normal tissues (Figure 6B,C). Furthermore, LUAD patients with low MTSS1 protein level owned a poor three-year survival rate relative to those with high MTSS1 protein level (Figure 6D). To explore the relationship between the co-expression of LINC00491 and the protein level of MTSS1 and three-year survival rates of LUAD patients, the LUAD patients were divided into four groups (LINC00491 High and MTSS1 High, LINC00491 High and MTSS1 Low, LINC00491 Low and MTSS1 High, and LINC00491 Low and MTSS1 Low) based on the median level of LINC00491 and MTSS1 protein level. The results revealed that LUAD patients with high LINC00491 and low MTSS1 level showed the poorest overall survival rates, while those with low LINC00491 and high MTSS1 level obtained the best overall survival rates (Figure 4E). Collectively, the negative correlation between MTSS1 expression and overall survival was proved, and the co-expression of LINC00491 and MTSS1 could predict clinical outcomes in patients with LUAD.

## 4. Discussion

Recent studies have revealed that lncRNAs are aberrantly expressed between diverse tumor types and normal tissues, which suggests their potential value in cancer diagnosis, monitoring, prognosis or response to molecular therapy. Lung adenocarcinoma (LUAD) is the major histopathological subtype of lung cancer, occupying 60% of NSCLC, and more than 40% of lung cancer [34]. Despite application of chemotherapy and immunotherapy in clinical treatments, 5-year overall survival is still less than 20% [35]. The dysregulation of gene expression including protein-coding genes and non-coding RNAs is characteristic of tumor pathogenesis. To date, various lncRNAs have been identified as being associated with LUAD, providing novel insights into the molecular mechanism in LUAD tumorigenesis and progression. In this study, we combined RNA-seq analysis of three pairs of advanced LUAD and matched normal lung tissue samples with the TCGA databases to screen the aberrantly expressed lncRNAs in LUAD. Our study found that LINC00491 expression level is significantly upregulated and associated with disease progression and poor clinical outcomes in LUAD patients, which could serve as a prognostic biomarker in LUAD.

Previous studies on lncRNAs showed that they are involved in various biological functions through different molecular mechanisms. Some lncRNAs link themselves to the transcription site to regulate the expression of a gene via interaction with proteins [36,37]. Some can also function as sponges for miRNA or regulate antisense mRNAs at the posttranscriptional level [38,39]. Additionally, recent studies revealed that some lncRNAs could regulate protein stability through RNA-protein interactions [40]. In this study, we identified the direct interaction between LINC00491 and MTSS1 using RNA pull-down and RIP assays. Truncation experiments further revealed the minimal essential region of LINC00491 required for interacting with MTSS1. Further experiments showed that LINC00491 decreased the protein level of MTSS1 by shortening its half-time, suggesting that the interaction between LINC00491 and MTSS1 could regulate the degradation of MTSS1. Then, the treatment of MG132 significantly upregulated the expression of MTSS1, which indicated LINC00491 may regulate the ubiquitination level of MTSS1. Subsequent studies verified that LINC00491 facilitated the ubiquitination level of MTSS1, implying that LINC00491 promotes MTSS1 degradation via the ubiquitin–proteasome degradation pathway. Collectively, LINC00491 promotes LUAD proliferation, migration and metastasis by binding directly to MTSS1, further promoting ubiquitination and degradation.

Previous studies reported MTSS1 as a tumor and/or metastasis suppressor whose expression is negatively correlated with poor prognosis in some solid tumors, while the specific mechanism of low MTSS1 expression in LUAD is little reported [25]. Here, we further verified that MTSS1 functions as a tumor suppressor by impairing cell proliferation, migration and metastasis [41], and large number of clinical samples were used to confirm that low expression of MTSS1 in LUAD is associated with poor survival. Previously, MTSS1 was reported that modulates lamellipodia formation by interacting with actin-associated proteins [17]. Recently, Wu et al. found that MTSS1 activates Wnt/β-catenin signaling by promoting the phosphorylation of GSK3β in ovarian cancer [19]. Aberrant activation of Wnt/β-catenin signaling was reported to promote invasion and metastasis of various cancers, especially LUAD. We then checked the correlation between LINC00491/MTSS1 and Wnt/β-catenin signaling, and our data suggested that the overexpression of LINC00491 could activate Wnt/β-catenin signaling in A549 and H1299 cell lines, while enforced expression of MTSS1 decreased the phosphorylation of GSK3β and β-catenin expression. As a result, we revealed that the LINC00491/MTSS1/Wnt/β-catenin signaling axis regulates cell proliferation, migration and metastasis in LUAD, and the expression of LINC00491 and MTSS1 is closely associated with clinical prognosis in LUAD.

Although our findings illuminate that LINC00491 activates Wnt/β-catenin signaling via promoting MTSS1 ubiquitin–proteasome degradation, it is currently not clear how LINC00491 expression increases in the LUAD. Some microRNAs were reported to regulate MTSS1 in other cancer types such as miR-96-5p in ovarian cancer and miR-23a in colorectal cancer [19,42]. In this study, we demonstrated the ubiquitination modification of MTSS1 and identified the RNA-protein interaction that affects the MTSS1 ubiquitination. The truncation experiment revealed the interaction region of LINC00491, while the specific type of linkages that form polyubiquitin chains and lysine ubiquitination site need further exploration.

## 5. Conclusions

In summary, our study revealed that LINC00491 expression is upregulated in LUAD and that high expression of LINC00491 is positively correlated with tumor metastasis and poor survival. LINC00491 interacts with MTSS1 directly to facilitate ubiquitination degradation. Consistently with previous findings that MTSS1 is a tumor/metastasis suppressor, our study reveals that the LINC00491/MTSS1 axis facilitates tumor malignant progression via activating Wnt/β-catenin signaling. This study further confirms the diversity and complexity of lncRNA-protein interactions, and suggests that the LINC00491/MTSS1 axis could be a potential target in LUAD diagnosis and therapeutic strategies.

## Data Availability

Please contact the corresponding authors for all data requests.

## Supplementary Materials

The following supporting information can be downloaded at: https://www.mdpi.com/article/10.3390/cells11233737/s1; Figure S1: Transfection efficiency of LINC00491 was confirmed using RT-qPCR. Figure S2: In vitro assays of shNC and shLINC00491 groups. Figure S3: Mass spectrum of MTSS1 in results. Figure S4: LiCI reversed the tumor malignance impaired by the LINC00491 knockdown. Figure S5: In vitro rescue experiments. Table S1: Clinicopathological features of LUAD patients in the study. Table S2: Clinicopathological features of three pairs of LUAD and normal tissues in RNA-sequencing. Table S3: List of primary antibodies used in this study.
