# Supplementary material for: LINC00491 Facilitates Tumor Progression of Lung Adenocarcinoma via Wnt/β-Catenin-Signaling Pathway by Regulating MTSS1 Ubiquitination
Source: Cells. 2022 Nov 23;11(23):3737. doi: 10.3390/cells11233737 (PMC9738320; doi:10.3390/cells11233737)
Supplement: Supplementary file 1 [file cells-11-03737-s001.zip › Supplementary figures.pdf]

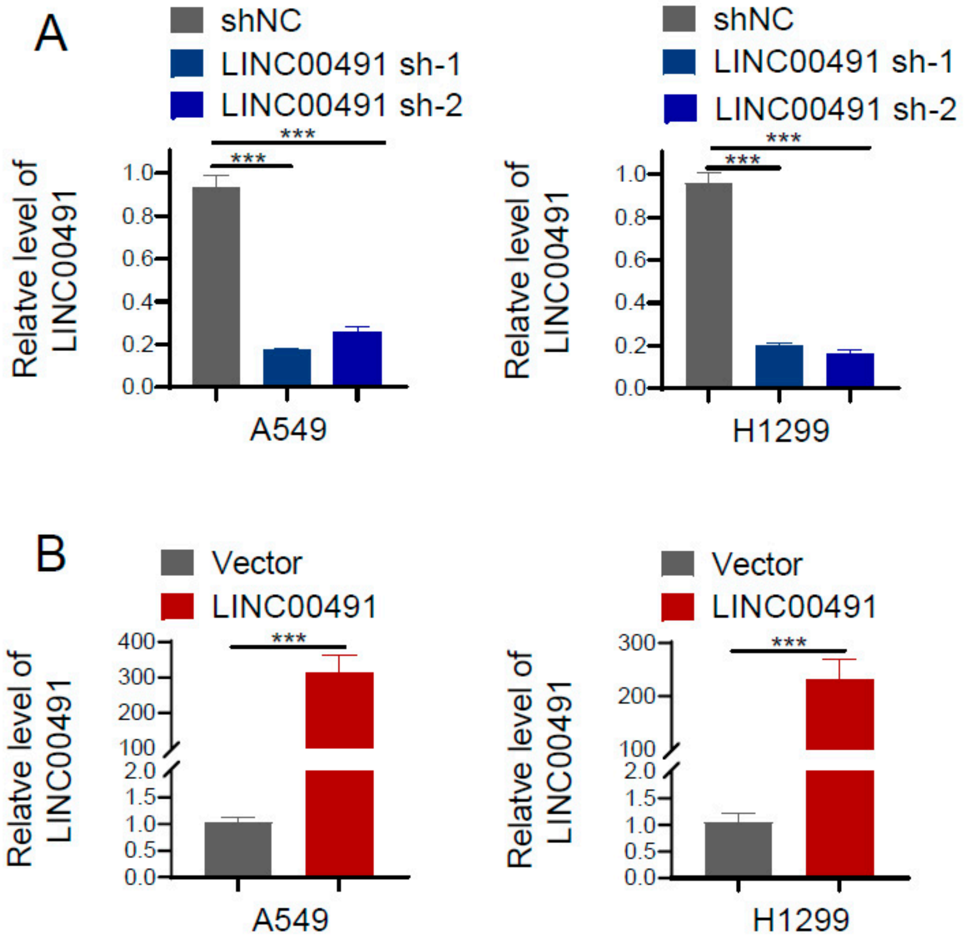

Figure S1. A RT-qPCR analysis of the LINC00491 in shNC and LINC00491 sh-1 and sh-2 groups in A549 and H1299 groups. **B** RT-qPCR analysis of the LINC00491 in Vector and LINC00491 groups in A549 and H1299 groups. In each experiment, three replicates were conducted. Data are presented as the mean ± SD. *, $P<0.05$; **, $P<0.01$; ***, $P<0.001$

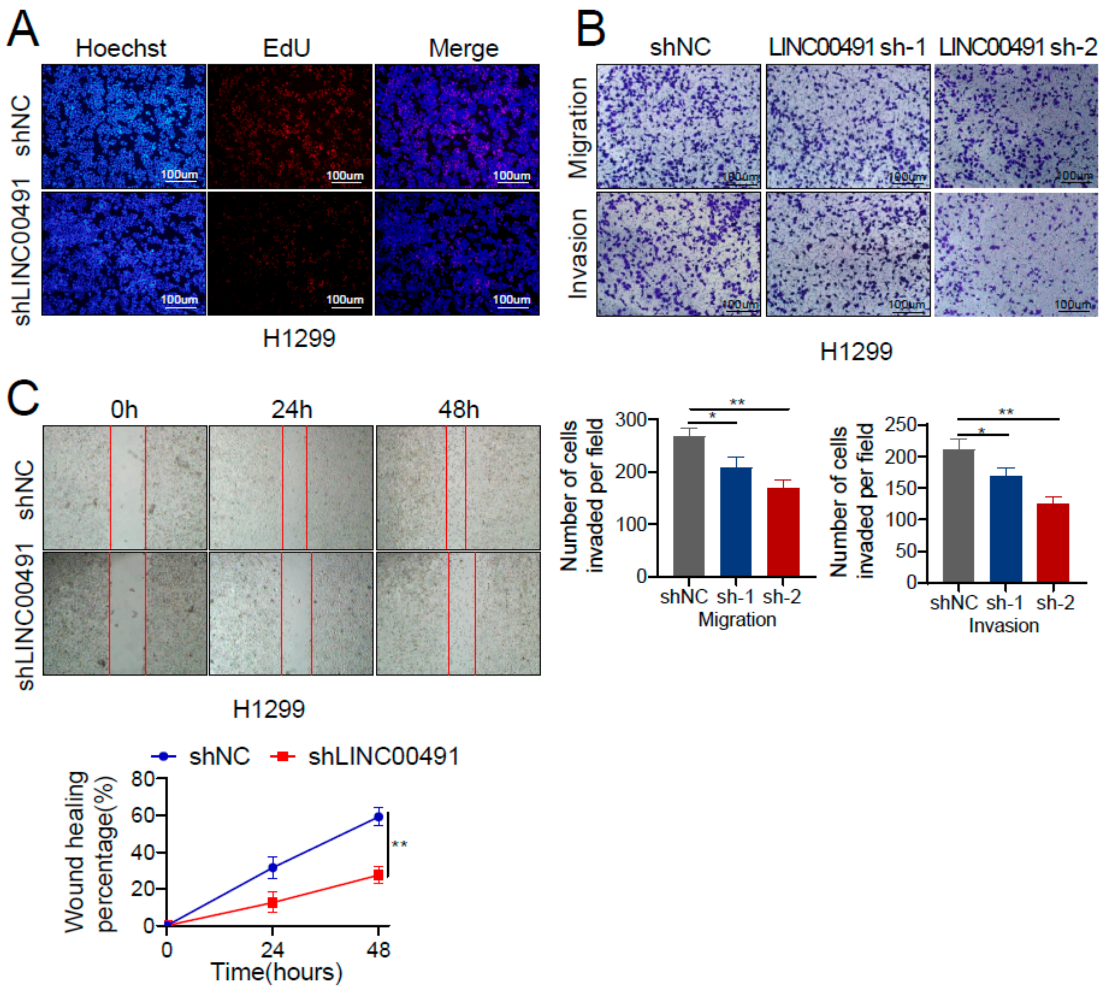

Figure S2. A The proliferation ability was measured by EdU assays in shNC and shLINC00491 groups. Scale bars=100 μm. **B** Transwell assays analyzed the migrative and invasive capacity of H1299 cells in shNC and LINC00491 sh-1 and sh-2 groups (upper panel). Quantification of results was exhibited on the bottom panel. Scale bars=100 μm. **C** Representative images of the scratch wound-healing assay in shNC and shLINC00491 groups (upper panel). Quantification of results was exhibited on the bottom panel. In each experiment, three replicates were conducted. Data are presented as the mean ± SD. *, $P<0.05$; **, $P<0.01$; ***, $P<0.001$

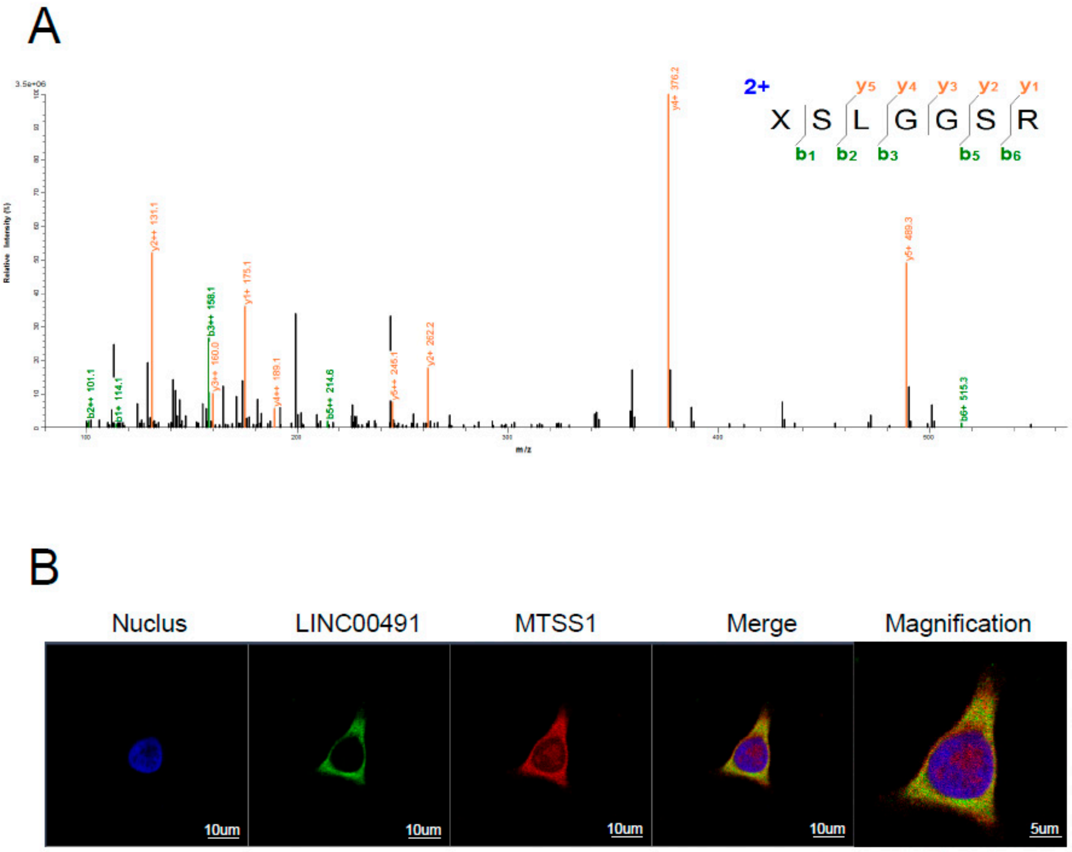

Figure S3. A LC-MS/MS analysis was conducted to identify proteins that interact with LINC00491 by analyzing immunoprecipitated proteins from A549 cells. **B** Representative images of the colocalization of LINC00491 with MTSS1 proteins were obtained by a confocal microscope, detected by FISH and IF assays in H1299 cells.

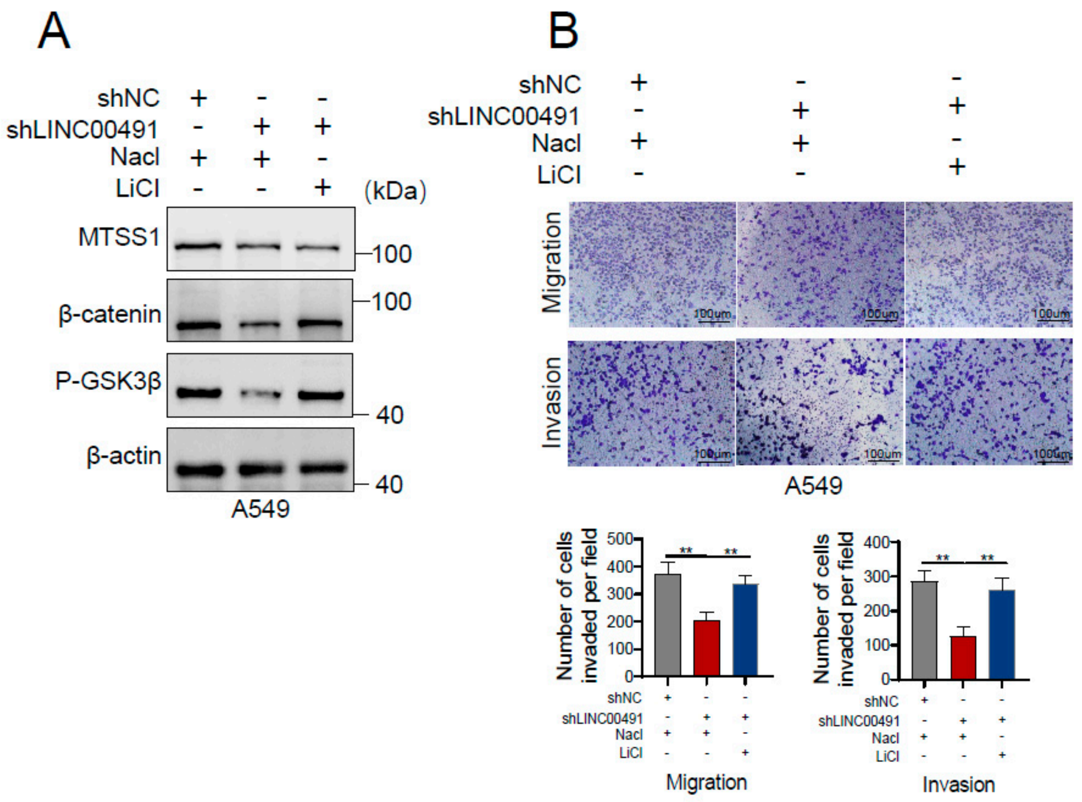

Figure S4. A Western blot analysis of the expression of β-catenin and p-GSK3β in LINC00491 knockdown or not A549 cells treated with LiCl or NaCl. **B** Transwell assays showed that Wnt agonist LiCI reversed the migrative and invasive capacity decreased by LINC00491 knockdown in A549 cells (upper panel). Quantification of results was exhibited on the bottom panel. Scale bars=100 μm.

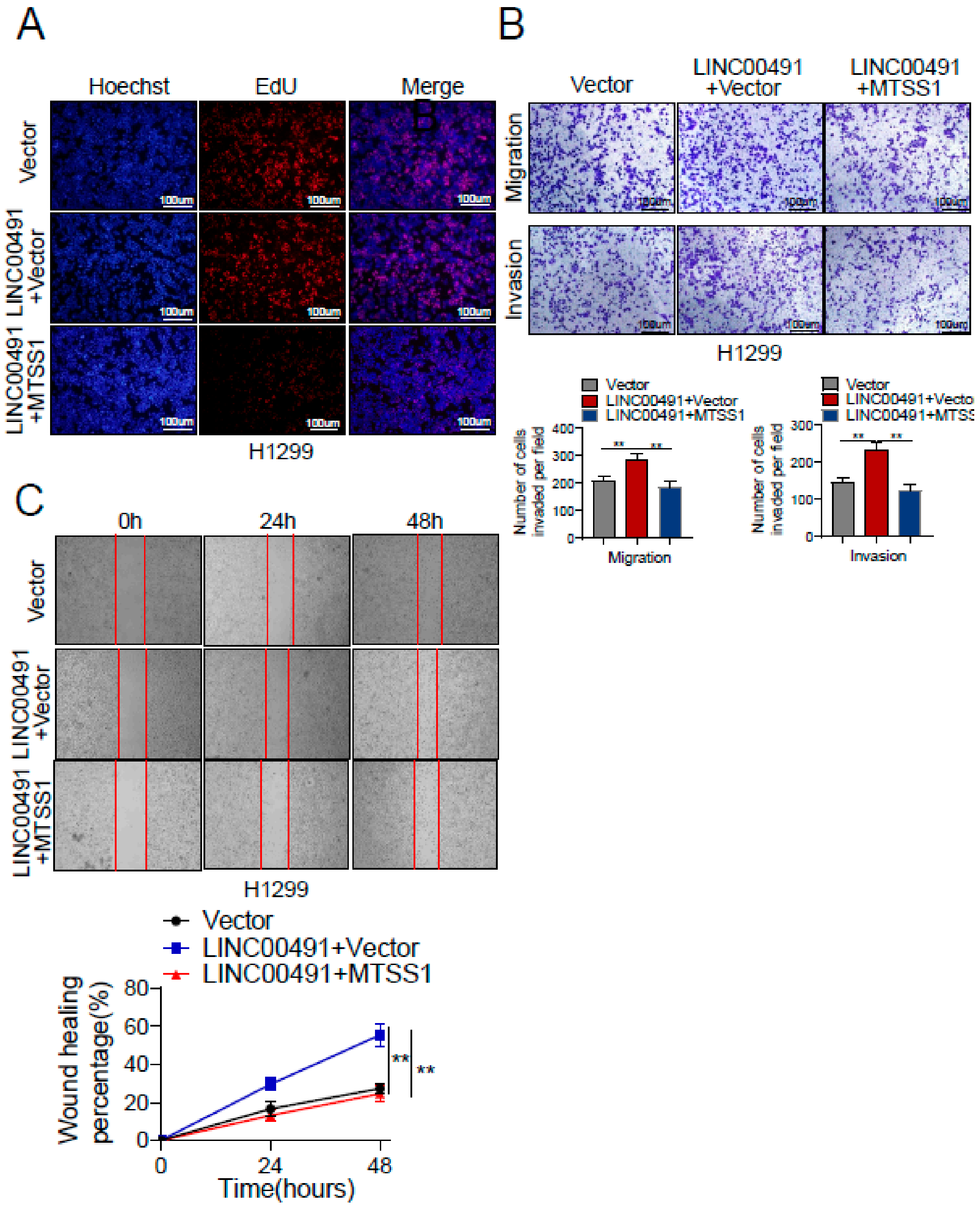

Figure S5. A The proliferation ability was measured by EdU assays showed that overexpression of MTSS1 reversed the growth-promotion effect of LINC00491 overexpression in H1299 cells. Scale bars=100 μm. **B** Transwell assays showed that overexpression of MTSS1 reversed the migrative and invasive capacity promoted by LINC00491 overexpression in H1299 cells (upper panel). Quantification of results was

exhibited on the bottom panel. Scale bars=100 μm. **C** Representative images of the scratch wound-healing assay showed that overexpression of MTSS1 reversed the migrative capacity promoted by LINC00491 overexpression in H1299 cells (upper panel). Quantification of results was exhibited on the bottom panel. In each experiment, three replicates were conducted. Data are presented as the mean ± SD. *, $P<0.05$; **, $P<0.01$; ***, $P<0.001$
