# Supplementary material for: LINC00491 Facilitates Tumor Progression of Lung Adenocarcinoma via Wnt/β-Catenin-Signaling Pathway by Regulating MTSS1 Ubiquitination
Source: Cells. 2022 Nov 23;11(23):3737. doi: 10.3390/cells11233737 (PMC9738320; doi:10.3390/cells11233737)
Supplement: Supplementary file 1 [file cells-11-03737-s001.zip › Supplementary table.pdf]

Table S1. Clinicopathological characteristics of 82 patients with LUAD

Characteristics		Number	No. of patients		P-value
			LINC00491 High	LINC00491 Low	
Age (y)	≥60	45	26	19	0.5016
	<60	37	15	22	
Gender	Male	47	27	20	0.2623
	Female	35	14	21	
pT status	1	21	8	13	0.2858
	2	26	14	12	
	3	24	11	13	
	4	11	8	3	
pN status	0	2	1	1	0.1047
	1	43	27	16	
	2	16	6	10	
	3	21	7	14	
TNM	II	38	17	21	0.5067
	III	44	24	20	

Table S2. Clinicopathological characteristics of 3 patients with LUAD

Case	Age (y)	Gender	pT status	pN status	TNM
1	53	Male	2	3	III
2	77	Male	3	2	III
3	73	Female	4	2	III

Table S3. Primary antibodies used in this study.

Source	Primary antibodies	Catalog no.
Cell Signaling Technology	Anti-MTSS1 antibody produced in rabbit	93065
Cell Signaling Technology	Anti-Ubiquitin antibody produced in rabbit	43124
Cell Signaling Technology	Anti-β-catenin antibody produced in rabbit	8480
Cell Signaling Technology	Anti-Phospho-GSK-3β antibody produced in rabbit	5558
ProteinTech	Anti-Beta Actin antibody produced in mouse	66009-1-Ig
